# Group‐Based Relational Savoring Intervention in Mothers of Young Children in Iran: Testing Impacts of Memory Type

**DOI:** 10.1111/famp.70092

**Published:** 2025-11-14

**Authors:** Bahar Sharifi, Zabihollah Kaveh Farsani, Jessica L. Borelli

**Affiliations:** ^1^ Department of Counseling Shahrekord University Shahrekord Iran; ^2^ Department of Psychology University of California Irvine USA

**Keywords:** maternal caregiving quality, parental satisfaction, relational savoring, safe haven, secure base

## Abstract

Relational savoring, a prevention/intervention approach that involves reflecting on positive moments of connection with another person, promotes wellbeing in the parenting relationship. In this program, parents savor memories with strong attachment content (including Secure Base or Safe Haven memories), but it is currently unknown whether the benefits of relational savoring differ as a function of memory type. Within a sample of Iranian mothers of children younger than 6, this study examines impacts of RS administered in Persian on closeness to child, parental satisfaction/competence, and maternal caregiving quality. In this randomized controlled trial, 150 mothers from a city in Iran were randomized into three 50‐member groups (relational savoring‐Safe Haven [RS‐Safe Haven]; relational savoring‐Secure Base [RS‐Secure Base]; Control Group). Participants completed assessments at pretest, posttest, and follow‐up (3 months post‐intervention). The two intervention groups (RS‐Safe Haven, RS‐Secure Base) participated in one‐hour group relational savoring sessions that met weekly for four weeks. The Control Group received no intervention. RS interventions significantly improved maternal closeness, parental satisfaction, and maternal sensitivity/availability compared to control, with gains evident at both post‐test and follow‐up. While both RS‐Secure Base and RS‐Safe Haven groups showed comparable improvements on most outcomes, RS‐Secure Base demonstrated stronger effects for parental efficacy, particularly at follow‐up. These findings have important implications for future work with relational savoring, suggesting that savoring both Secure Base and Safe Haven memories can enhance maternal outcomes.

## 1 Introduction

Across the globe, most adults become parents at some point in their lives (Roser et al. 2025), and most regard parenthood as one of the most significant roles they will undertake (Pew 2023). A transformational experience, parenthood also presents considerable emotional challenges (Kerr et al. 2020; Nelson et al. 2014). Parenting young children demands a high level of emotional regulation, requiring parents to remain emotionally steady in the face of rapidly shifting and often intense emotional expressions from their children, who rely heavily on caregivers to support their regulation (Hajal and Paley 2020; Rutherford et al. 2015). Within a single day, a toddler or preschool‐aged child may cycle through a range of emotional states—throwing a tantrum in frustration, squealing in joy, clinging in fear, or falling asleep in a parent's arms. Parents often journey with their children through these emotional peaks and valleys, offering comfort, co‐regulation, and shared joy.

At day's end, caregivers often reflect on a broad spectrum of emotionally salient parenting moments that vary in both valence and intensity. A parent might dwell on a moment of distress, such as feeling ineffective when unable to soothe a child who is experiencing a tantrum, which could contribute to feelings of inadequacy or helplessness (van IJzendoorn et al. 2004). Conversely, they may recall a moment of successful attunement, such as comforting a fearful child or sharing in their excitement, which can reinforce their sense of competence and strengthen the parent–child bond. The emotional demands and rewards of parenting are closely intertwined; navigating a young child's emotional highs and lows offers both challenges and opportunities to cultivate meaning, purpose, and connection in the parent–child relationship (Booth et al. 2021).

Mothers and fathers both play essential roles in children's development, though their contributions are often distinct (Nelson‐Coffey et al. 2019). Mothers engage in direct, intimate contact with their children beginning in the prenatal period, when early attachment processes are thought to emerge. These interactions shape developmental trajectories and often establish mothers as primary sources of physical and emotional security (Azimifar et al. 2018; da Rosa et al. 2021; Della Vedova et al. 2023; Villalobos 2014). Unlike many Western societies, where most attachment research has been conducted, contexts such as Iran and other Middle Eastern cultures are shaped by traditional gender roles that place mothers at the center of caregiving. Within these cultural frameworks, motherhood is framed as a role requiring devotion and self‐sacrifice (Modiri et al. 2024; Mousavi 2020). While such expectations underscore the value of maternal care, they can also generate stress and reduce opportunities for self‐care (Rizzo et al. 2013). Interventions that guide mothers to reflect on moments of closeness and competence may therefore help sustain caregiving while restoring the emotional rewards of parenting.

## 2 Relational Savoring: A Program to Enhance Focus on Moments of Positive Connection

Relational savoring (RS; Borelli 2024; Borelli, Smiley, et al. 2020, 2024) is a brief, strengths‐based prevention or intervention program that is designed to help people focus on moments of positive connection occurring in their daily lives. Most extensively studied in the realm of parenting, RS involves helping a parent focus deeply on a memory of a time when they provided comfort to their child (safe haven memory; Ainsworth et al. 2015; Bowlby 1982) or a time when they supported their child in doing something they might not have been able to do without their guidance (secure base memory; Ainsworth et al. 2015; Bowlby 1982). For instance, a parent completing an RS intervention may focus on a time when she helped her child achieve a goal, such as climbing on top of a big rock, focusing on the way her child was looking at her with a mix of fear (at first), then excitement, pride, a sense of accomplishment, and the confidence of knowing her mother was there supporting her in trying something new.

RS is informed by two theoretical frameworks: attachment theory (Bowlby 1973) and the broaden‐and‐build theory of positive emotions (Fredrickson 2001). From an attachment perspective, early parent–child interactions are viewed as foundational for later development (Bowlby 1973). To foster a sense of security, children require caregivers who are attentive, responsive, and invested in their growth (Ainsworth 1967; Bowlby 1973). Thus, enhancing parents' sensitivity and positive engagement with their child is critical. At the same time, parenting inevitably involves challenging interactions that can undermine parents' confidence, highlighting the value of strategies that help parents focus on moments of felt connection and competence. By anchoring in these positive experiences, parents can strengthen their confidence in the caregiving role and sharpen their attention to their child's needs. The broaden‐and‐build theory of positive emotions (Fredrickson 2001) provides a complementary lens. It posits that positive emotional states expand perspective and foster creativity, enabling individuals to consider new possibilities. Within the context of RS, this broadened state creates an optimal foundation for relationship‐based interventions, as it enhances parents' openness and receptivity to their child's thoughts and feelings.

RS has been implemented within several populations. This study focuses on the in‐person delivery of the intervention with parents of young children. In this format, RS begins with a brief mindfulness exercise, followed by memory selection. During this phase, an intervener helps the mother recall positive, attachment‐rich memories involving her child. The intervention emphasizes two main categories of memories—Safe Haven and Secure Base—which align with Ainsworth's (1967) conceptualization of sensitivity. *Safe Haven* memories involve times when the child was distressed (e.g., sad or frightened) and the mother provided comfort, fostering closeness and security. *Secure Base* memories reflect moments when the mother supported the child's exploration, growth, or development (e.g., helping the child learn to tie their shoe). If a mother cannot recall a memory from either category, the intervener encourages her to focus on another moment of positive connectedness that evoked feelings of closeness with the child. After discussing two or three candidate memories—whether Safe Haven, Secure Base, or Positive Connectedness—the intervener and mother collaboratively select one memory to focus on during the next phase of the intervention: memory reflection. In this phase, the intervener guides the participant through structured prompts designed to deepen her re‐experiencing of the memory and facilitate meaning‐making from it.

### 2.1 Outcomes Targeted by Relational Savoring

RS is believed to influence a parent's affect, cognition, and behavior. In terms of affect, it is theorized to enhance parents' emotional connection with their child by encouraging them to focus on moments of closeness. By fostering this focus, RS helps parents perceive a stronger bond with their child, which is significant as greater parental closeness is a predictor of long‐term relationship quality (Lin and Hammersmith 2024; Zhai et al. 2024). Moreover, a close or bonded parent–child relationship has been associated with a reduced long‐term impact of stressful life events on children (Ge et al. 2009). Conversely, low parental closeness is associated with heightened risk for child abuse and neglect (Chen et al. 2024) and has been linked to risk for psychiatric symptoms in adulthood (Kidd et al. 2022).

RS is also thought to impact cognition by enhancing parents' awareness of how their sensitive parenting behaviors influence their child both in the moment and over time. This process is believed to strengthen parents' sense of competence, which encompasses both efficacy—the ability to solve parenting challenges—and satisfaction, or the pleasure derived from fulfilling their parental role (Jones and Prinz 2005; Ohan et al. 2000). A heightened sense of parental competence is crucial, as parents who feel more competent tend to be more responsive and receptive to their children (Dumka et al. 2010), as well as engage in fewer negative parenting behaviors (Sanders and Woolley 2005), perceive their children as less defiant (Coleman and Karraker 2003), and exhibit fewer aggressive, disruptive, and controlling behaviors (Fang et al. 2021; Knerr et al. 2013).

Finally, RS is thought to shape behavior by directing parents' attention to instances in which they effectively met their child's needs and encouraging them to reflect on the positive emotions associated with these moments. This reflection fosters a greater awareness of their child's needs, reinforces the satisfaction of meeting those needs, and strengthens their motivation to respond sensitively in the future. Sensitivity, as conceptualized by Ainsworth (1982), consists of two key components: responsiveness—the parent's attunement to and ability to meet the child's needs—and availability—their psychological and physical presence in supporting the child. Enhancing parental sensitivity is critical, as it is a key predictor of children's attachment security (Ainsworth et al. 2015; Bakermans‐Kranenburg et al. 2003; De Wolff and Van Ijzendoorn 1997).

Research trials of RS have generally supported its theoretical model (Borelli 2024; Borelli, Smiley, et al. 2020, 2022), although not all components of the model have been tested. RS trials have been conducted in various formats. The most extensive RS trials have been conducted in individual, in‐person sessions (Borelli et al. 2022; Borelli et al. 2019; Borelli, Yates, et al. 2020; Wang et al. 2020), but other studies have involved an internet‐based administration of RS (Burkhart et al. 2015; Borelli et al. 2014; Lord et al. 2025), also revealing impacts on targeted outcomes. Recent and ongoing studies have involved administering RS through an *m*health application (Nguyen et al. 2025) and through Zoom sessions (Arcos et al. 2023). Although most studies of RS have involved sessions administered to an individual, RS has been adapted into a group format (Ansarifar et al. 2025; Borelli, Yates, et al. 2020).

Compared to an active control condition, personal savoring (savoring a personal experience), RS increases feelings of closeness to child, positive emotion, and parenting sensitivity (Borelli et al. 2022). Further, among Latina mothers, RS increases reflective functioning and uptake of the intervention (Borelli et al. 2022). Research conducted with other populations suggests that RS increases relationship satisfaction among long‐distance romantic relationship partners (Borelli et al. 2014), increases help‐seeking among adults with depression (Straszewski and Siegel 2018), reduces attachment anxiety among adolescent males (Wang et al. 2020), and reduces physiological reactivity (Borelli et al. 2019) and reduces grief (Basic and Bryant 2025) among bereaved older adults.

One significant area of need is in the cross‐cultural expansion of RS. To date, most RS studies have been conducted in the United States. Looking beyond RS, the majority of attachment‐based interventions and attachment research studies have been conducted in the United States and Western nations (Chen 2015; Maciel 2023; van IJzendoorn and Kroonenberg 1988). Ansarifar et al. (2025) recently reported on their findings of the only study to administer RS in person in another cultural context. In a sample of mothers of children under the age of 5 in Iran, they administered RS in a group therapy context to mothers, comparing this approach to a no‐treatment control. The mothers assigned to the RS group exhibited improvements in closeness to child and maternal availability at post‐treatment and two‐month follow‐up, while not having impacts on parenting competence and sensitivity/responsiveness (Ansarifar et al. 2025). This initial study of RS in Iran provided preliminary evidence of its efficacy compared to a no‐treatment control, which suggests the need for future exploration of this intervention approach.

### 2.2 Memory Type in Relational Savoring

Although research demonstrates that RS yields positive outcomes, less is known about which components are most essential to its effectiveness. One element that has drawn attention is the type of memory used in the savoring process. Prior work suggests that some memories are more vulnerable to “spoiling”—when positive recollections become tinged with negative emotion (Borelli 2024; Gaskin 2021). Spoiling is counterproductive in RS, where the goal is to amplify positive affect and strengthen meaning in moments of connection. Clinical observations indicate that spoiling occurs more often with Safe Haven memories than with Secure Base memories (Borelli 2024; Gaskin 2021). This may reflect the negativity bias, whereby individuals tend to dwell on negative aspects of experiences even when positive elements are present (Vaish et al. 2008; Lazarus 2021). Thus, for some parents, the negative emotions embedded in Safe Haven memories may overshadow the positive focus intended by RS.

Yet there is evidence that recalling positive memories can buffer stress, dampening cortisol responses and reducing negative affect (Speer et al. 2017), and that positively reframing adverse memories can recast their emotional tone, enhancing resilience and future positivity (Speer et al. 2021). These findings illustrate the tension between the risks of spoiling and the therapeutic promise of savoring. In RS, clients generate their own memories, which may have Safe Haven or Secure Base content, making it essential to examine whether memory type produces differential effects. Safe Haven memories, in particular, often involve comforting a distressed child. For instance, a parent might recall soothing a child who has gotten hurt. While the reflection can highlight the parent's competence and the child's reliance on them, it may also evoke intrusive thoughts of fear, anxiety, or guilt, which disrupt savoring. Still, Safe Haven memories are often rich in attachment‐related meaning and emotionally resonant content (Borelli 2024), suggesting they may be especially valuable for intervention despite their vulnerability to spoiling. While Secure Base memories may involve worry for the child who takes a risk in trying something new (e.g., first day at a new school), they are more likely to involve joy and excitement (Gaskin 2021).

To date, no studies have explored the impact of memory type on savoring outcomes. Research is needed to clarify whether memory type moderates intervention outcomes. Addressing this gap not only advances RS research but also contributes to attachment science more broadly. Both Safe Haven and Secure Base experiences are central to attachment‐based interventions such as Circle of Security (Hoffman et al. 2006; Powell et al. 2013). Understanding how parents respond emotionally to these different memory types may guide intervention design and enrich our understanding of the psychological experience of parenting (e.g., Hajal and Paley 2020).

## 3 Current Investigation

The present study advances knowledge on RS in two ways. First, using a randomized controlled trial, we replicate prior work by testing the effectiveness of RS in group therapy sessions with parents of children age six and under in Iran—an understudied context for RS and attachment‐based interventions more broadly. To extend earlier findings, this second Iranian trial was conducted in a different region (a city in Kurdestan province) and assessed both previously examined outcomes and an additional outcome, parenting satisfaction. Participants were randomly assigned to RS or a waitlist control, and outcomes were measured at post‐treatment and at a 3‐month follow‐up. We hypothesized that, compared to controls, parents in the RS condition (referred to as RS‐Combined for this analysis) would show greater improvements in (a) affective outcomes (e.g., closeness to child), (b) cognitive outcomes (e.g., parental competence, parenting satisfaction), and (c) behavioral outcomes (e.g., parental sensitivity).

Second, this study explores mechanisms of RS by examining whether the type of memory targeted during the intervention influences outcomes. Specifically, we compared the effects of savoring Secure Base versus Safe Haven memories on affective, cognitive, and behavioral outcomes, relative to one another and to the control group. Given the limited prior research, we advanced no specific hypothesis regarding memory type, treating this as an exploratory aim.

## 4 Method

### 4.1 Participants and Procedures

All the mothers with children under six years of age in a city in Iran's Kurdestan province were eligible to participate. Inclusion criteria included (a) being a biological mother to a child under 6 and (b) having a child who resides with the mother full‐time. The exclusion criteria included (a) concurrent involvement in individual or group therapeutic sessions at the same time as the research study, (b) having a history of chronic mental health disorders (e.g., major depressive disorder), and (c) taking psychotropic medicine. In 2024, 150 of these women were selected through volunteering‐purposive sampling. Mothers interested in participating underwent eligibility screening and then completed informed consent for participation in the study. Mothers who had more than one child were asked to select one child within the targeted age range (under 6) to focus on throughout the study. Using a random number generator, the participants were then divided randomly into one of two experimental/RS groups (Safe Haven, Secure Base) or a control group. Participants who missed an intervention session (*n* = 5) were dropped from the study. All study procedures, including group sessions and self‐report measures, were administered in Persian, the native language of the participants.

Mothers provided informed consent for participation in the study. As for the members of the control group, they were ensured that they could receive the intervention if they completed the research process. The studied groups were also ensured of the confidentiality. The study was approved at Shahrekord University with the ethics code of IR.SKU.REC.1403.043.

Before (pre‐test) and after the intervention phase (post‐treatment) as well as during the follow‐up period 3 months later (follow‐up), participants completed an assessment battery. After the completion of the study, a one‐day workshop was held for the control group to train them on the savoring Secure Base and Safe Haven memories.

### 4.2 Relational Savoring Intervention Protocol

Participants assigned to the RS condition were placed in groups of 5‐to‐7 individuals for intervention delivery. The intervention followed a structured protocol consisting of four one‐hour group sessions, held once weekly. During each session, participants completed a RS exercise. The Secure Base group recalled and savored Secure Base memories, and the Safe Haven group recalled Safe Haven memories. Participants in the control group were assigned to a no‐treatment condition, receiving no intervention.

The RS protocol was adapted from previous research (Ansarifar et al. 2025; Borelli 2024; Borelli, Smiley, et al. 2020, 2022), modified for both a group setting and the Iranian cultural context. The original group‐based format (Ansarifar et al. 2025) was co‐developed over several months by the RS developer and Iranian psychologists to align with Iranian cultural values while preserving the RS protocol's core principles. This culturally responsive format, designed to enhance participant comfort, was replicated here with one modification: separating groups into RS‐Secure Base and RS‐Safe Haven.

A key innovation of our study was the modification that enabled a direct, experimental comparison between the two memory types. In prior RS interventions, participants were free to select which memories to savor—Secure Base, Safe Haven, or a combination of both—introducing variability that made it difficult to determine the unique effects of each memory type. Our design addresses this limitation by experimentally isolating memory type, providing a clearer test of which components of RS are most impactful. The intervention dosage—four weekly one‐hour sessions—was aligned with the length of the most extensive prior studies.

The interveners, who consisted of one female with a master's degree and one male with a doctorate in counseling, conducted the groups in pairs. Each pair of interveners co‐facilitated all sessions for a single group, ensuring consistency and minimizing the potential for facilitator‐related bias across sessions. The two intervention groups (RS‐Secure Base and RS‐Safe Haven) were each led by the same pair of facilitators. Within the groups, each participant individually recalled and documented a positive memory in detail using a written form, which was then submitted to the facilitator. Following this period of individual reflection, participants were given the opportunity to share their memories and experiences with the group.

At the conclusion of each session, participants were asked to observe and document moments of closeness/connection with their children, which would then be discussed during the following session. Each new session began with participants providing feedback, offering an opportunity to reflect on the effects of the savoring exercises. With participants' consent, all sessions were audio‐recorded for research purposes.

Interveners guided the mothers through the following steps: (1) Mindfulness relaxation Exercise: Following prior research (Borelli et al. 2022), each session began with a minute‐long mindfulness breathing exercise designed to enhance sensory awareness and increase openness to the intervention. (2) Psychoeducation: The intervener introduced concepts such as attachment and mindfulness, using accessible language; (3) Memory Selection: The intervener facilitated the process of recalling meaningful attachment‐related memories. Priority was given to memories that reflected attachment security. Participants assigned to the Secure Base group focused on instances where the mother helped her child achieve a goal (e.g., try something new, take a risk, explore a new environment) while providing emotional support, while participants assigned to the Safe Haven group focused on instances where the mother provided comfort and reassurance in times of distress; and (4) Memory Reflection: Participants engaged in a structured five‐step reflection process (Borelli 2024): (a) Sensory Reflection: Recalling and vividly re‐experiencing sensory details, (b) Emotion Focus: Identifying, labeling, and expressing the bodily sensations and emotions associated with the recalled memory, (c) Meaning Making: Reflecting on the significance of the memory for oneself, the child, and the relationship, (d) Future Focus: Considering how the savored experience might shape future interactions with the child, and (e) Mind‐Wandering: Allowing spontaneous thoughts related to the memory, self, or child to emerge for deeper processing.

At the end of each session, participants were asked to pay attention to moments of closeness with their child throughout the week and encouraged to remember them for discussion in the next session. From the second week onward, each session began with a brief period for participants to share their weekly experiences, thoughts, and feelings regarding their child. Participant feedback was recorded for further reflection.

### 4.3 Measures

#### 4.3.1 Closeness to Child

Mothers completed the Inclusion‐of‐Other‐in‐Self Scale (IOS; Aron et al. 1992) Child Version, a single‐item measure that evaluates the degree of closeness between the respondent and another individual. Participants select from seven pairs of circles that vary in overlap, with one circle labeled “self” and the other labeled “other.” Responses range from 1 (*no overlap*) to 7 (*complete overlap*), indicating increasing levels of closeness. The IOS correlates highly with other measures of relationship closeness (Gächter et al. 2015). Previous studies (Borelli et al. 2022; Sichko et al. 2016) have used the IOS‐Child Version to examine parent–child closeness. Notably, Ansarifar et al. (2025) used the Persian version in Iran.

#### 4.3.2 Parenting Sense of Competence

Mothers completed the Parenting Sense of Competence Scale (Gibaud‐Wallston and Wandersmann 1978, PSOC), a 16‐item questionnaire including two subscales: satisfaction, which measures frustration, anxiety, and motivation related to parenting, and efficacy, which assesses the parent's confidence in problem‐solving. The measure is scored on a 6‐point Likert scale (1 = *completely disagree* to 6 = *completely agree*). This measure has been previously validated for use in Iran (Omidvar and Liraviani Njad 2019). In this study, Cronbach's *α* for the efficacy and satisfaction subscales was acceptable (pre‐treatment: 0.76 and 0.72, post‐treatment: 0.71 and 0.73, and follow‐up: 0.77 and 0.75), respectively.

#### 4.3.3 Parenting Satisfaction

Mothers completed the Kansas parental satisfaction (Schumm and Hall 1994), a 3‐item scale scored using a 7‐point Likert scale, from 1 (*highly dissatisfied*) to 7 (*highly satisfied*). The scores on the three items were summed to obtain a total score for parental satisfaction. Mousavi (2023) used the scale in an Iranian sample (*α*: 0.82). In this study, *α* was acceptable: pre‐treatment: 0.83, post‐treatment: 0.87, and follow‐up: 0.84.

#### 4.3.4 Maternal Sensitivity

Mothers completed the maternal Caregiving Quality Scale (Ghanbari et al. 2012). This scale includes 32 items assessing (a) conflict and confusion, (b) sensitivity and responsiveness, and (c) availability, rated on a 5‐point Likert scale, from 1 (*never correct*) to 5 (*always correct*). This questionnaire has previously established content validity, face validity, and criterion validity in an Iranian sample (Ghanbari et al. 2012). In this study, we focused on the sensitivity/responsiveness and availability subscales because they were most centrally related to RS—reliability (*α*) was acceptable (pre‐treatment: 0.73 and 0.71, post‐treatment: 0.77 and 0.75, and follow‐up: 0.72 and 0.70), respectively.

### 4.4 Data Analytic Plan

First, we evaluated descriptive statistics and correlations among our central study variables. Next, we tested Hypotheses 1–3 by collapsing the two experimental groups (RS: Safe Haven, RS: Safe Haven) into a single group, referred to as RS‐Combined for the purpose of these analyses, and comparing those against the control group using an analysis of covariance (ANCOVA). Then we tested Research Questions 1–3 by conducting a multivariate ANCOVA in which we compared the three groups (RS: Safe Haven, RS: Secure Base, control) on all outcome variables at the three time points.

To assess the adequacy of the sample size (*N* = 150, 50 per group) for testing Hypotheses 1–3, a post hoc power analysis was conducted following Shieh (2020; using the Superpower package in R). Assuming a medium effect size (*η* ^2^ = 0.06, based on the observed group effect for IOS Closeness to Child), *α* = 0.05, and three groups across three timepoints, the analysis yielded power > 0.80, confirming sufficient statistical power to detect significant differences in the ANCOVA models.

## 5 Results

Prior to testing hypotheses, we confirmed that the data met ANCOVA assumptions. Additionally, one‐way ANOVA analyses did not reveal any significant pre‐test differences between groups (Secure Base, Safe Haven, Control) for any outcome variables (all *p* > 0.05). Pre‐test scores were included as covariates in the ANCOVA models to control for baseline variations. Table 1 presents descriptive statistics for the sample.

**TABLE 1:** Means (SD) of outcome variables by group and timepoint.

Variable	Group	Pre‐test	Post‐test	Follow‐up
IOS closeness to child	Safe Haven	4.17 (1.56)	4.92 (1.29)	5.61 (1.02)
	Secure Base	4.70 (1.47)	5.76 (1.04)	5.88 (0.92)
	Control	5.00 (1.70)	5.00 (1.67)	4.94 (1.67)
	Combined	4.44 (1.52)	5.34 (1.17)	5.75 (0.97)
PSOC satisfaction	Safe Haven	3.35 (0.85)	3.79 (0.73)	3.96 (0.65)
	Secure Base	3.55 (0.79)	3.84 (0.79)	3.99 (0.76)
	Control	3.29 (0.80)	3.33 (0.85)	3.39 (0.86)
	Combined	3.45 (0.82)	3.82 (0.76)	3.98 (0.71)
PSOC efficacy	Safe Haven	4.05 (0.88)	4.09 (0.80)	4.35 (0.64)
	Secure Base	4.21 (0.96)	4.40 (0.85)	4.54 (0.79)
	Control	4.29 (0.84)	4.26 (0.85)	4.29 (0.88)
	Combined	4.13 (0.92)	4.25 (0.83)	4.45 (0.72)
KPS parental satisfaction	Safe Haven	3.54 (1.14)	5.44 (0.83)	6.14 (0.52)
	Secure Base	4.62 (1.19)	5.57 (0.92)	6.01 (0.63)
	Control	5.08 (1.29)	5.02 (1.24)	5.01 (1.24)
	Combined	4.08 (1.17)	5.51 (0.88)	6.08 (0.58)
MCS sensitivity and responsiveness	Safe Haven	4.09 (0.63)	4.53 (0.37)	4.64 (0.22)
	Secure Base	4.29 (0.58)	4.59 (0.33)	4.62 (0.27)
	Control	4.03 (0.76)	4.01 (0.80)	4.00 (0.79)
	Combined	4.19 (0.61)	4.56 (0.35)	4.63 (0.25)
MCS availability	Safe Haven	4.11 (0.53)	4.71 (0.28)	4.76 (0.21)
	Secure Base	4.39 (0.51)	4.74 (0.18)	4.73 (0.19)
	Control	4.17 (0.69)	4.19 (0.68)	4.20 (0.71)
	Combined	4.25 (0.52)	4.73 (0.23)	4.75 (0.20)

*Note:* Each group (Secure Base, Safe Haven, Control) initially included 50 mothers; 5 participants were dropped due to missing an intervention session, resulting in *N* = 45 per group (*N* = 135 total). Pre‐test assessments were conducted before the intervention, post‐test immediately after the four‐week intervention, and follow‐up three months post‐intervention.

Abbreviation: SD, standard deviation.

### 5.1 Hypothesis 1: Predicting Changes in Closeness to Child

#### 5.1.1 Differences Between RS‐Combined and Control

**FIGURE 1:**
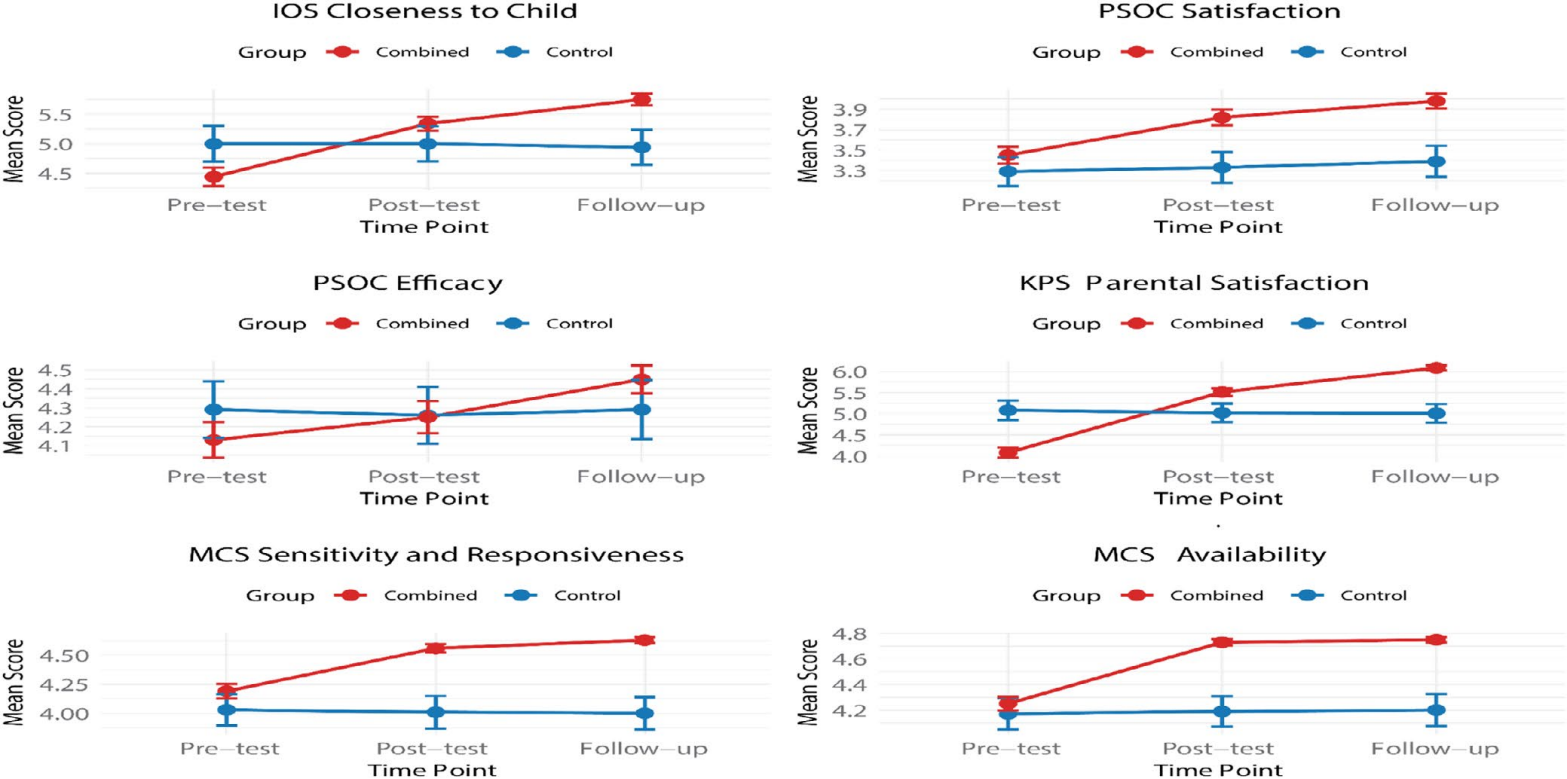
Differences in outcomes between RS‐Combined and Control.

**FIGURE 2:**
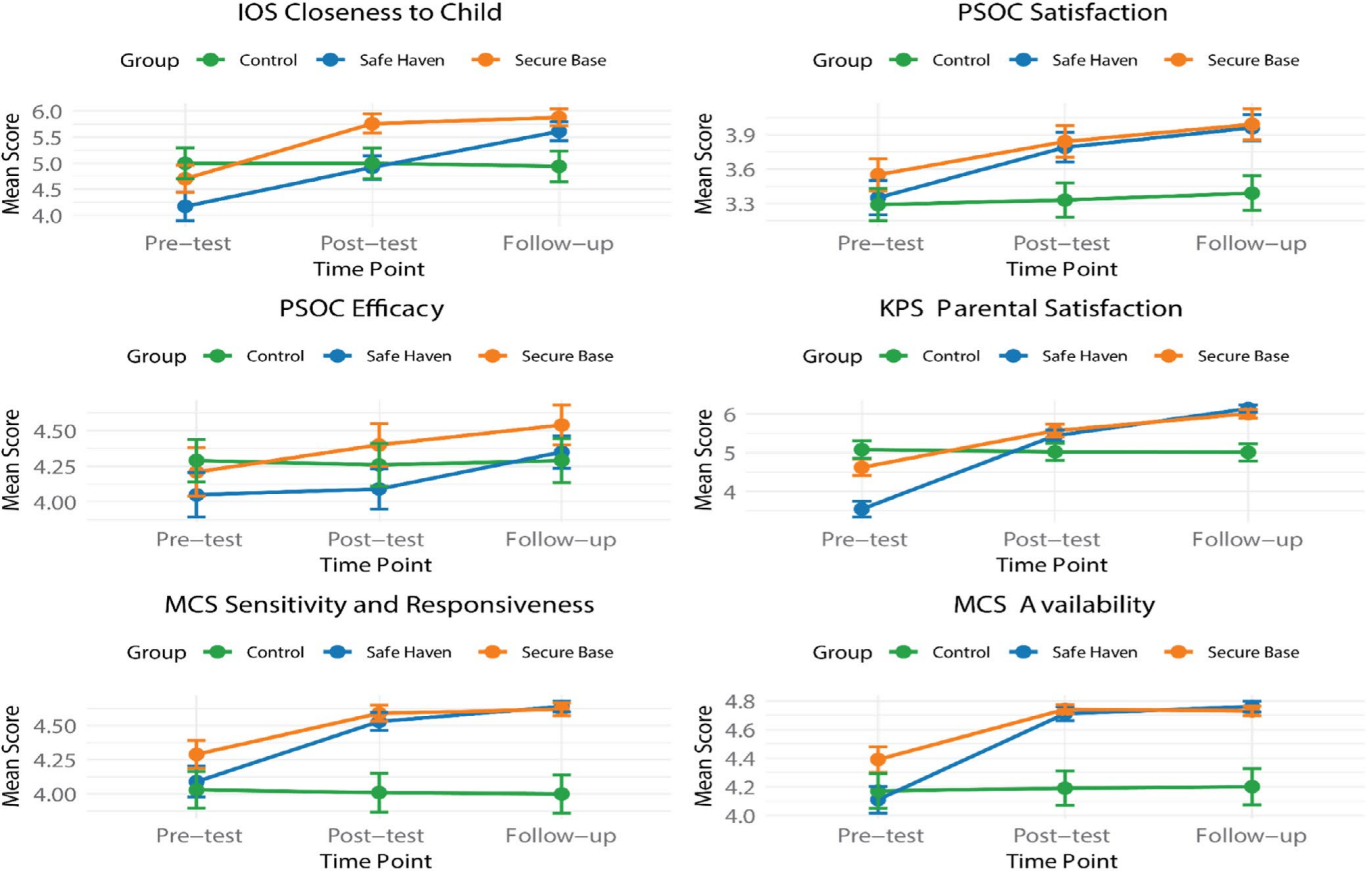
Differences in outcomes between Control, RS‐Safe Haven, and RS‐Secure Base.

An ANCOVA (see Table 2, Figures 1 and 2) revealed that the RS‐Combined variable and the experimental time point (pre‐test, post‐test, and follow‐up) had significant effects on closeness to the child (*F* = 14.059, *p* = 0.002 for group; *F* = 6.42, *p* < 0.001 for time point). The group factor explained 8.5% of the variance in closeness to child scores, indicating a moderate effect of the intervention on parental closeness. The RS‐Combined group significantly outperformed the Control group in IOS at both post‐test (mean difference = 0.604, *p* = 0.001) and follow‐up (mean difference = 0.853, *p* < 0.001). RS‐Combined's mean increased from ~4.44 (average of Secure Base: 4.70, Safe Haven: 4.17) to 5.34 (average of Secure Base: 5.76, Safe Haven: 4.92) at post‐test (change = 0.90) and to 5.75 (average of Secure Base: 5.88, Safe Haven: 5.61) at follow‐up (change = 1.31), compared to the Control group's negligible change (0.00 at post‐test, −0.06 at follow‐up; pre‐test: M = 5.00, post‐test: M = 5.00, follow‐up: M = 4.94). These results confirm significant improvements in maternal closeness at both timepoints (see Tables 2 and 3).

**TABLE 2:** Analyses of covariance.

**IOS closeness to child (Hypothesis 1)**				**PSOC satisfaction (Hypothesis 2)**			
**Effect**	***F***	***p***	***η*** ^**2**^	**Effect**	***F***	***P***	***η*** ^**2**^
Model	10.239	0.000	0.085	Model	11.59	0.000	0.095
Fixed effect	5726.39	0.000	0.928	Fixed effect	6276.19	0.000	0.95
Group	14.059	0.002	0.028	Group	14.00	0.000	0.06
Experimental timepoint	6.42	0.000	0.060	Experimental timepoint	9.19	0.000	0.40
**PSOC efficacy (Hypothesis 2)**				**KPS parental satisfaction (Hypothesis 2)**			
**Effect**	***F***	***p***	***η*** ^**2**^	**Effect**	***F***	***P***	***η*** ^**2**^
Model	2.711	0.030	0.024	Model	17.91	0.000	0.139
Fixed effect	11575.65	0.000	0.963	Intercept	10616.05	0.000	0.960
Group	3.08	0.047	0.010	Group	5.29	0.005	0.121
Experimental timepoint	2.34	0.098	0.40	Experimental timepoint	30.52	0.000	0.025
**Sensitivity and responsiveness (Hypothesis 3)**				**Availability (Hypothesis 3)**			
**Effect**	***F***	***p***	***η*** ^**2**^	**Effect**	***F***	***P***	***η*** ^**2**^
Model	20.38	0.000	0.156	Model	25.69	0.000	0.189
Intercept	24735.19	0.000	0.982	Intercept	34882.72	0.000	0.987
Group	30.73	0.000	0.122	Group	29.97	0.000	0.119
Experimental timepoint	10.03	0.000	0.043				

**TABLE 3:** ANCOVA post hoc tests for timepoint and group comparisons.

Variable	Timepoint comparisons			Group comparisons			
	Pre‐T and Post‐T	Pre‐T and FU	Post‐T and FU	CO and SH	CO and SB	SH and SB	CO and CM
**IOS**							
Mean Diff	0.604	0.853	0.248	0.595	0.971	0.376	0.20
*p*	0.001	< 0.001	0.402	< 0.001	< 0.001	0.009	0.042
**PSOC satisfaction**							
Mean Diff	0.263	0.386	0.123	0.459	0.366	0.093	0.41
*p*	0.013	< 0.001	0.543	< 0.001	< 0.001	0.938	0.028
**PSOC efficacy**							
Mean Diff	0.063	0.206	0.143	0.266	0.236	0.235	0.02
*p*	1.000	0.106	0.434	0.032	0.034	0.049	0.88
**KPS parental satisfaction**							
Mean Diff	0.604	0.853	0.248	0.466	0.548	0.082	0.19
*p*	0.001	< 0.001	0.402	0.015	0.003	1.000	0.036
**MCS sensitivity and responsiveness**							
Mean Diff	0.237	0.276	0.042	0.491	0.409	0.082	0.45
*p*	0.001	< 0.001	1.000	< 0.001	< 0.001	0.669	0.19
**MCS availability**							
Mean Diff	0.429	0.339	0.095	0.366	0.336	0.030	0.39
*p*	< 0.001	< 0.001	0.316	0.011	0.023	1.000	0.03

*Note:* Each group (Safe Haven, Secure Base, Control) initially included 50 mothers; 5 participants were dropped due to missing an intervention session, resulting in *N* = 45 per group (*N* = 135 total).

Abbreviations: CM, RS‐Combined; CO, Control; FU, Follow‐up; Mean Diff, Mean Difference; Post‐T, Post‐test; Pre‐T, Pre‐test; SB, Secure Base; SH, Safe Haven.

#### 5.1.2 Differences Between Three Conditions

Both RS groups (Secure Base and Safe Haven) exhibited significantly greater increases in IOS Closeness to Child scores from pre‐test to post‐test (mean difference = 0.604, *p* = 0.001) and follow‐up (mean difference = 0.853, *p* < 0.001) compared to the Control group, which showed minimal change (pre‐test: M = 5.00, SD = 1.70; post‐test: M = 5.00, SD = 1.67; follow‐up: M = 4.94, SD = 1.67). Specifically, RS‐Secure Base showed significant increases from pre‐test (M = 4.70, SD = 1.47) to post‐test (M = 5.76, SD = 1.04; mean difference vs. Control = 0.595, *p <* 0.001) and follow‐up (M = 5.88, SD = 0.92; mean difference vs. Control = 0.971, *p* < 0.001). The RS‐Safe Haven group showed a substantial increase from pre‐test (M = 4.17, SD = 1.56) to post‐test (M = 4.92, SD = 1.29) and follow‐up (M = 5.61, SD = 1.02; mean difference vs. Control = 0.971, *p* < 0.001), though the difference compared to the Control group was statistically significant only at follow‐up. No differences were revealed between RS‐Secure Base and RS‐Safe Haven for IOS at any time point (*p* = 0.009), indicating that both RS interventions had comparable effects on closeness. However, the RS‐Secure Base group had significantly higher IOS scores than the Control group at both post‐test and follow‐up while the RS‐Safe Haven group only had significantly higher IOS scores than the Control group at follow‐up (see Table 3).

### 5.2 Hypothesis 2: Predicting Changes in Parental Competence and Satisfaction

#### 5.2.1 Parental Competence

##### 5.2.1.1 Differences Between RS‐Combined and Control

In terms of PSOC Satisfaction, RS‐Combined significantly outperformed the Control group at post‐test (mean difference = 0.263, *p* = 0.013) and follow‐up (mean difference = 0.386, *p <* 0.001). The RS‐Combined group's mean increased from ~3.33 (Control's pre‐test mean) to 3.82 at post‐test (change = 0.49) and to 3.98 at follow‐up (change = 0.65), compared to the Control group's minimal change (0.00 at post‐test, 0.06 at follow‐up; pre‐test: M = 3.33, post‐test: M = 3.33, follow‐up: M = 3.39). The RS‐Combined intervention significantly enhanced parental satisfaction at both timepoints.

In terms of PSOC Efficacy, the RS‐Combined group showed no significant difference from the Control group at post‐test (mean difference = 0.063, *p* = 1.000) or follow‐up (mean difference = 0.206, *p* = 0.106). The RS group's mean changed from ~4.26 to 4.25 at post‐test (change = −0.01) and to 4.45 at follow‐up (change = 0.19), compared to the Control group's minimal change (0.00 at post‐test, 0.03 at follow‐up; pre‐test: M = 4.26, post‐test: M = 4.26, follow‐up: M = 4.29). This indicates limited impact on efficacy.

##### 5.2.1.2 Differences Between Three Conditions

Next we examined differences across the three groups in satisfaction and efficacy. The RS‐Secure Base group's PSOC Satisfaction score increased from 3.33 to 3.84 (change = 0.51) at post‐test and 3.99 (change = 0.66) at follow‐up, significantly exceeding the Control group's negligible change (0.00 post‐test, 0.06 follow‐up; mean differences = 0.459 and 0.366, *p <* 0.001). The RS‐Safe Haven group's score rose from ~3.33 to 3.79 (change = 0.46) at post‐test and 3.96 (change = 0.63) at follow‐up. No differences existed between RS groups (*p* = 0.938).

In terms of efficacy, we found that the RS‐Secure Base group's PSOC Efficacy score increased from 4.26 to 4.40 (change = 0.14) at post‐test (not significant) and 4.54 (change = 0.28) at follow‐up (mean difference = 0.266, *p* = 0.032), while the RS‐Safe Haven group's score dipped to 4.09 (change = −0.17) at post‐test but rose to 4.35 (change = 0.09) at follow‐up (mean difference = 0.236, *p* = 0.034). RS‐Secure Base outperformed RS‐Safe Haven at follow‐up (*p* = 0.049).

#### 5.2.2 Parental Satisfaction

##### 5.2.2.1 Differences Between RS‐Combined and Control

The RS‐Combined group significantly outperformed the Control group at post‐test (mean difference = 0.604, *p* = 0.001) and follow‐up (mean difference = 0.853, *p <* 0.001). The RS‐Combined group's mean increased from ~5.02 to 5.51 (average of Secure Base: 5.57, Safe Haven: 5.44) at post‐test (change = 0.49) and to 6.08 (average of Secure Base: 6.01, Safe Haven: 6.14) at follow‐up (change = 1.06), compared to the Control group's negligible change (0.00 at post‐test, −0.01 at follow‐up; pre‐test: M = 5.02, post‐test: M = 5.02, follow‐up: M = 5.01). These results confirm significant improvements in parental satisfaction at both timepoints.

##### 5.2.2.2 Differences Between Three Conditions

The RS‐Secure Base group's KPS score increased from 5.02 to 5.57 (change = 0.55) at post‐test and 6.01 (change = 0.99) at follow‐up (mean differences = 0.466, *p* = 0.015; 0.548, *p* = 0.003), while the RS‐Safe Haven group's score rose to 5.44 (change = 0.42) at post‐test and 6.14 (change = 1.12) at follow‐up (mean difference = 0.19, *p* = 0.036 in combined analysis). No differences existed between RS groups (*p* = 1.000), and both outperformed the Control group's minimal change (0.00 post‐test, −0.01 follow‐up).

### 5.3 Hypothesis 3: Predicting Changes in Maternal Sensitivity

#### 5.3.1 Differences Between RS‐Combined and Control

In terms of sensitivity/responsiveness, the RS‐Combined group significantly outperformed the Control group at post‐test (mean difference = 0.237, *p* = 0.001) and follow‐up (mean difference = 0.276, *p* < 0.001). The RS‐Combined group's mean increased from ~4.01 to 4.56 at post‐test (change = 0.55) and to 4.63 at follow‐up (change = 0.62), compared to the Control group's negligible change (0.00 at post‐test, −0.01 at follow‐up; pre‐test: M = 4.01, post‐test: M = 4.01, follow‐up: M = 4.00). The RS‐Combined effect was marginally significant (mean difference = 0.45, *p* = 0.19), but individual timepoint analyses confirm robust improvements.

In terms of availability, the RS‐Combined group significantly outperformed the Control group at post‐test (mean difference = 0.429, *p* < 0.001) and follow‐up (mean difference = 0.339, *p <* 0.001). The RS group's mean increased from ~4.19 to 4.73 (average of Secure Base: 4.74, Safe Haven: 4.71) at post‐test (change = 0.54) and to 4.75 (average of Secure Base: 4.73, Safe Haven: 4.76) at follow‐up (change = 0.56), compared to the Control group's minimal change (0.00 at post‐test, 0.01 at follow‐up; pre‐test: M = 4.19, post‐test: M = 4.19, follow‐up: M = 4.20). The RS‐Combined RS effect was significant (mean difference = 0.39, *p* = 0.03), confirming enhanced maternal availability at both timepoints.

#### 5.3.2 Differences Between Three Conditions

The RS‐Secure Base group's MCS Sensitivity score increased from ~4.01 to 4.59 (change = 0.58) at post‐test and 4.62 (change = 0.61) at follow‐up (mean differences = 0.491 and 0.409, *p <* 0.001), while the RS‐Safe Haven group's score rose to 4.53 (change = 0.52) at post‐test and 4.64 (change = 0.63) at follow‐up (mean difference = 0.45, *p* = 0.19 in combined analysis). No differences existed between RS groups (*p* = 0.669), and both outperformed the Control group's negligible change (0.00 post‐test, −0.01 follow‐up), though RS‐Safe Haven's effect was marginal.

The RS‐Secure Base group's MCS Availability score increased from ~4.19 to 4.74 (change = 0.55) at post‐test and 4.73 (change = 0.54) at follow‐up (mean differences = 0.366, *p* = 0.011; 0.336, *p* = 0.023), while the RS‐Safe Haven group's score rose to 4.71 (change = 0.52) at post‐test and 4.76 (change = 0.57) at follow‐up (mean difference = 0.39, *p* = 0.03 in combined analysis). No differences existed between RS groups (*p* = 1.000), and both significantly outperformed the Control group's minimal change (0.00 post‐test, 0.01 follow‐up).

### 5.4 Follow‐Up Analyses

As a robustness check, an intent‐to‐treat (ITT) analysis was conducted using multiple imputation to include all 150 randomized participants, accounting for the 5 dropped due to missing an intervention session. The ITT results, presented in the Supporting Information, were consistent with the primary ANCOVA findings, confirming significant improvements in the RS groups (RS‐Secure Base and RS‐Safe Haven) compared to the Control group across outcome variables, with no significant differences between RS‐Secure Base and RS‐Safe Haven for most outcomes.

As an extra step to account for the group‐based nature of the interventions, where within‐group variation may differ from between‐group variation, a multilevel modeling analysis was conducted as a robustness check. This analysis modeled parents nested within intervention groups (RS‐Secure Base, RS‐Safe Haven, Control) across three timepoints (pre‐test, post‐test, follow‐up), with random effects for groups and fixed effects for condition and timepoint. Results, presented in the Supporting Information, align with the primary ANCOVA findings. Results are presented by outcome variable.

## 6 Discussion

Given the paramount importance of parent–child relationships in fostering children's socioemotional development, identifying ways to bolster parents in their efforts is critical. The current study reports on the second examination of RS conducted in Iran, and just the third test of RS outside the United States. This study also represents one of the few attachment‐based interventions to be tested outside of the Western world and in the Middle East, contributing to a growing body of knowledge about the utility of this approach within this cultural context (Maciel 2023). Given the centrality of mother–child relationships within Iran (e.g., Modiri et al. 2024), and the pressure mothers face (Rizzo et al. 2013), there is a pressing need to develop parenting programs to support their well‐being and the well‐being of their children (Mousavi 2020). We discuss the findings that emerged from this initial trial and their implications below.

This study replicated the findings of Ansarifar et al. (2025), which documented positive outcomes of RS when delivered in Iran—the first such examination in the Middle East. In the former study, RS was delivered in a group format, a novel approach at the time. Consistent with prior work (Ansarifar et al. 2025; Borelli et al. 2022; Burkhart et al. 2015; Lord et al. 2025), participants assigned to the RS‐Combined group showed greater increases in perceived closeness to their child than those in the control group. This was a moderate effect size, explaining 8.5% of the variance in closeness. Revisiting emotionally positive memories may strengthen the bond mothers have with their children, shaping the maternal representation of the relationship. This is important, as closeness is linked to reduced risk of abuse and neglect (Chen et al. 2024) and stronger parent–child relationship quality (Jones and Prinz 2005). RS‐Combined also led to greater increases in parenting satisfaction on the PSOC and the KPS, replicating results from a US group‐based RS intervention (Borelli, Yates, et al. 2020). Finally, RS increased maternal sensitivity, aligning with previous studies (Ansarifar et al. 2025; Borelli, Kerr, et al. 2022; Smiley et al. 2024). As maternal sensitivity strongly predicts children's attachment security (De Wolff and Van Ijzendoorn 1997), it remains a key target in parenting interventions. Importantly, RS‐Combined did not impact parenting efficacy—there was no significant difference between the intervention group and the control group—which is unfortunate given the important role efficacy plays in parenting behaviors (Coleman and Karraker 2003; Fang et al. 2021; Knerr et al. 2013). Researchers interested in targeting parental efficacy as an outcome may wish to focus RS in a specific way to target efficacy (for example, by using Secure Base RS), or use other interventions. Taken together, however, this second study of RS in Iran provides a strong replication of the first study, demonstrating impacts on many similar parenting outcomes as were observed in Ansarifar et al. (2025), heightening confidence in RS specifically, and attachment‐based interventions more generally, as a useful approach for improving parenting wellness in Iranian mothers.

The second set of analyses explored differences in outcomes between the Secure Base and Safe Haven RS groups. These analyses were prompted by prior concerns that Safe Haven memories might be more susceptible to “spoiling,” where participants focus on negative aspects of relationships, a phenomenon observed in clinical case studies (Borelli 2024) and explained by the negativity bias (Vaish et al. 2008). However, findings revealed minimal differences between the two groups. Both groups outperformed the Control condition in most cases. The only significant difference between the two groups was that mothers in the RS‐Secure Base group reported higher parenting efficacy than those in the RS‐Safe Haven group at follow‐up. This may be due to Secure Base memories emphasizing moments when mothers supported their child's independence, reinforcing a sense of parenting efficacy. Conversely, RS‐Safe Haven memories may better support feelings of nurturance. The fact that the groups were not significantly different in the other outcomes suggests that savoring Safe Haven memories does not reduce overall intervention effectiveness. Notably, the quality of the savoring process was not assessed, so potential spoiling effects remain unclear. These findings support the use of either memory type in RS, depending on which is more salient—rich in detail, positive emotion, and attachment content.

These findings have implications for the delivery of RS as well as attachment‐based interventions in general. When it comes to RS, the implications are that interveners should focus on whichever memory generates the greatest feelings of positive emotion and connectedness in the participant, rather than ensuring that participants savor Secure Base or Safe Haven memories. In future work, it will be interesting to examine whether savoring specific types of attachment memories has targeted impact on specific types of sensitivity (e.g., whether savoring Safe Haven memories increases availability and comfort provision while savoring Secure Base increases autonomy promotion). This could have implications for other intervention programs that seek to strengthen Safe Haven or Secure Base behaviors in parents (e.g., Hoffman et al. 2006). For instance, RS‐Secure Base may be indicated for parents who suffer from low parenting efficacy or who struggle to support their child's autonomy, whereas RS‐Safe Haven may be indicated for parents who demonstrate relative deficits in comfort provision.

### 6.1 Strengths and Limitations

As only the second study of its kind conducted in the Middle East, this research makes a meaningful contribution to the literature. Moreover, it is the first to experimentally investigate the differential effects of RS‐Secure Base versus RS‐Safe Haven, offering valuable insights for future intervention development and attachment research. A notable strength of the study is the inclusion of both post‐treatment and three‐month follow‐up assessments, allowing for the evaluation of the intervention's sustained effects. This work contributes to a growing body of research focused on identifying low‐cost, scalable interventions designed to enhance parental well‐being and the quality of parent–child relationships.

The study's contributions should be viewed considering its limitations. Most notably, it used a no‐treatment control group. While this was appropriate for evaluating the novel group‐based format of RS, future research should compare RS to an active treatment—such as personal savoring, mindfulness‐based stress reduction, or parent education—to more rigorously assess its efficacy. Second, the sample size was small and replication with a larger sample is important in order to enhance confidence in the findings. Third, due to budgeting limitations, the outcome measures were exclusively self‐report measures. In future studies, it will be important to examine observed behaviors (e.g., observed measures of maternal sensitivity) to gain external viewpoints of the constructs of interest. Further, our primary interest in this early study was to seek to improve maternal well‐being, but ultimately an additional goal is to impact children's well‐being. The current study did not assess any child outcomes—future work would enhance our understanding of the impacts of RS by including measures of children's emotion regulation, distress tolerance, emotion understanding, and attachment to the parent. In addition, to promote group cohesion, the protocol in the current study was to drop members who missed a single group. However, this resulted in the exclusion of 5 participants from the group and from analysis. In this study, we did not collect outcome data on these mothers, but in future studies, we will collect outcome data on them to have a complete intent‐to‐treat sample. Finally, due to our desire to have a homogenous sample, we focused exclusively on mothers in this study, who are primary caregivers in this cultural context. However, fathers also serve an important role in children's lives and may also benefit from parenting interventions such as this one. In future studies, it will be important to develop ways to engage fathers in programs in which they can reflect on their parenting experiences.

## 7 Conclusion

This study contributes important new knowledge to the literature on RS by providing additional evidence regarding its efficacy in a Middle Eastern context and offering cross‐cultural validation of earlier findings. The intervention was successful in improving affective, cognitive and behavioral outcomes among mothers of young children. Notably, the study provides the first experimental evidence that the type of attachment memory—whether Secure Base or Safe Haven—does not differentially influence intervention outcomes. These findings suggest that both memory types are viable targets in intervention design. As part of a broader effort to develop scalable, cost‐effective strategies to support parental well‐being and parent–child relationship quality, this research lays the groundwork for further exploration of RS in diverse populations and contexts.

## Conflicts of Interest

The authors declare no conflicts of interest.

## Supporting information


**Appendix S1:** famp70092‐sup‐0001‐AppendixS1.docx.

## Data Availability

The data that support the findings of this study are available from the corresponding author upon reasonable request.
