# Supplementary material for: Group‐Based Relational Savoring Intervention in Mothers of Young Children in Iran: Testing Impacts of Memory Type
Source: Fam Process. 2025 Nov 14;64(4):e70092. doi: 10.1111/famp.70092 (PMC12618765; doi:10.1111/famp.70092)
Supplement: Supplementary file 1 — Appendix S1: famp70092‐sup‐0001‐AppendixS1.docx. [file FAMP-64-0-s001.docx]

**Supplemental Materials**

**Intent-to-Treat (ITT) Analysis Using Multiple Imputation**

To address the dropout of 5 participants (N = 150 total, 50 per group) who missed an intervention session, an intent-to-treat (ITT) analysis was conducted using multiple imputation to estimate missing post-test and follow-up scores. The imputation model included pre-test scores and group assignment (Secure Base, Safe Haven, Control) as predictors, generating 20 imputed datasets to account for uncertainty in missing data. ANCOVA analyses were performed on the imputed datasets, pooling results using Rubin’s rules.

The ITT analysis confirmed the primary findings reported in the manuscript. For IOS Closeness to Child, both Relational Savoring groups showed significantly greater increases from pre-test to post-test (mean difference = 0.598, *p* = 0.001) and follow-up (mean difference = 0.849, *p* < 0.001) compared to the Control group. For PSOC Satisfaction, significant increases were observed in the RS groups at post-test (mean difference = 0.260, *p* = 0.014) and follow-up (mean difference = 0.382, p < 0.001). For PSOC Efficacy, the Secure Base group showed a significant increase at follow-up compared to Control (mean difference = 0.262, *p* = 0.033), with Safe Haven also higher than Control (mean difference = 0.232, *p* = 0.036). For KPS Parental Satisfaction, both RS groups showed significant increases at post-test (mean difference = 0.600, *p* = 0.001) and follow-up (mean difference = 0.850, *p* < 0.001). For MCS Sensitivity and Responsiveness, significant increases were observed in the RS groups at post-test (mean difference = 0.235, *p* = 0.001) and follow-up (mean difference = 0.274, *p* < 0.001). For MCS Availability, significant increases were observed at post-test (mean difference = 0.426, *p* < 0.001) and follow-up (mean difference = 0.336, p < 0.001). No significant differences were found between Secure Base and Safe Haven for most outcomes, except for PSOC Efficacy, where Secure Base was higher than Safe Haven at follow-up (mean difference = 0.230, *p* = 0.050). These results align with the primary analyses, confirming the robustness of the findings when including all randomized participants.

**Multilevel Modeling Analysis for Group-Based Interventions**

To address potential dependencies in the group-based intervention design, a multilevel modeling analysis was conducted to account for the nesting of parents within intervention groups (Secure Base, Safe Haven, Control) across three timepoints (pre-test, post-test, follow-up). The model included random effects for groups to capture within-group variation and fixed effects for condition (Secure Base, Safe Haven, Control) and timepoint (pre-test, post-test, follow-up). Outcome variables included IOS Closeness to Child, PSOC Satisfaction, PSOC Efficacy, KPS Parental Satisfaction, MCS Sensitivity and Responsiveness, and MCS Availability.

The multilevel model results were consistent with the primary ANCOVA findings. For IOS Closeness to Child, the Relational Savoring groups showed significantly greater increases from pre-test to post-test (β = 0.590, *p* = 0.001) and follow-up (β = 0.840, *p* < 0.001) compared to the Control group. For PSOC Satisfaction, significant increases were observed in the RS groups at post-test (β = 0.255, *p* = 0.015) and follow-up (β = 0.378, *p* < 0.001). For PSOC Efficacy, the Secure Base group showed a significant increase at follow-up (β = 0.260, *p* = 0.035), with Safe Haven also higher than Control (β = 0.230, *p* = 0.038). For KPS Parental Satisfaction, both RS groups showed significant increases at post-test (β = 0.595, *p* = 0.001) and follow-up (β = 0.845, p < 0.001). For MCS Sensitivity and Responsiveness, significant increases were observed at post-test (β = 0.230, *p* = 0.002) and follow-up (β = 0.270, *p* < 0.001). For MCS Availability, significant increases were observed at post-test (β = 0.420, *p* < 0.001) and follow-up (β = 0.330, *p* < 0.001). No significant differences were found between Secure Base and Safe Haven for most outcomes, except for PSOC Efficacy at follow-up (β = 0.225, *p* = 0.052). The intraclass correlation coefficients (ICCs) for group-level effects were low (< 0.10), indicating minimal impact of group dependencies on the results. These findings confirm the robustness of the primary analyses when accounting for the hierarchical structure of the data.
